# Barriers and facilitators to diet, physical activity and lifestyle behavior intervention adherence: a qualitative systematic review of the literature

**DOI:** 10.1186/s12966-023-01424-2

**Published:** 2023-02-14

**Authors:** Alysha L. Deslippe, Alexandra Soanes, Celeste C. Bouchaud, Hailee Beckenstein, May Slim, Hugues Plourde, Tamara R. Cohen

**Affiliations:** 1 Faculty of Land and Food Systems, Food, Nutrition and Health, the University of British Columbia, Vancouver, Canada; 2 Healthy Starts, British Columbia Children’s Hospital Research Institute, Vancouver, Canada; 3 School of Exercise Science, Physical & Health Education, University of Victoria, Victoria, Canada; 4 PERFORM Research Centre, Concordia University, Montreal, Canada; 5 School of Human Nutrition, McGill University, Montreal, Canada

**Keywords:** Diet, Activity, Barriers, Facilitators, Intervention adherence, Behavior change, Qualitative methods

## Abstract

**Background:**

Consuming a balanced diet and regular activity have health benefits. However, many adults have a difficult time adhering to diet and activity recommendations, especially in lifestyle interventions. Adherence to recommendations could be improved if common facilitators and barriers are accounted for in intervention design. The aim of this systematic review was to understand perceived barriers and facilitators to lifestyle (diet and/or activity) intervention guidelines.

**Methods:**

This review follows the Preferred Reporting Items for Systematic Reviews and Meta-Analysis guidelines. Studies included relied on qualitative methods to explore the barriers and facilitators healthy adults ($\ge$ 18 years) experienced in lifestyle interventions. Google Scholar, Cochrane Reviews, Medline, PubMed, and Web of Science were searched from January 2005 to October 2021. Main themes from each paper were thematically analyzed and reported as a barrier or facilitator to adherence at the individual, environment or intervention level using inductively derived themes. Study quality was assessed using the Critical Appraisal Skills Programme.

**Results:**

Thirty-five papers were included. Of these, 46% were conducted in North America and the majority had more female participants (86% in mixed-sex studies, 26% females only). Similar themes emerged across all three levels as facilitators and barriers. At the individual level, attitudes, concern for health and physical changes. At the environmental level, social support, social accountability, changeable and unchangeable aspects of the community. Finally, delivery and design and content at the intervention level. An additional facilitator at the intervention level included fostering self-regulation through Behavior Change Taxonomies (BCT).

**Conclusions:**

Lifestyle interventions that foster self-regulatory skills, opportunities for social engagement and personalization of goals may improve behaviour adherence. This can be achieved through inclusion of BCT, tapering off of intervention supports, identification of meaningful goals and anticipated barriers with participants.

## Background

Eating a variety of nutrient rich foods and regularly being active contributes to positive psychological and physical health outcomes [1–4]. However, many adults do not meet dietary [5–7] or activity [8–10] guidelines. A 2020 systematic review of vegetable intake in 162 countries suggested that 88% of adults consumed less than the recommended 240 g of vegetables per day [5]. Furthermore, over a quarter of adults fail to meet the recommendations to participate in 150 min of moderate-intensity or 75 min of vigorous-intensity physical activity per week [10]. The discrepancy between ‘ideal’ practice of health behaviors and reality suggests a need for more effective strategies to support the practice of beneficial diet and activity behaviors [5, 10].

Lifestyle interventions are one avenue to support behavior change [11, 12]. Historically, these interventions have tended to focus on diet or activity related practices, though it is recognized that sleep and sedentary habits are also important [7, 13]. Lifestyle interventions can be rooted in a variety of frameworks and methods [14, 15], making it difficult to determine what components or ‘active ingredients’ [14–16] within interventions offer merit for behavior outcomes [17–19]. Literature has called for greater clarity in intervention reporting [14, 16], including the use of common terminology to describe *what* is occurring within an intervention to support behavior change, or *how* it’s delivered [14, 20]. One strategy to overcome this is to explore how participants within lifestyle interventions feel they are supported or hindered to change their behaviors [15]. By identifying perceived facilitators and barriers across diverse interventions and consolidating patterns from participants’ experiences [21–24], strategies to promote positive behavior change, regardless of intervention framework, aims or terminology can be revealed [15].

Interventions targeting behavior change do not act in isolation. Instead, they overlap with participant’s personal characteristics and structures in place surrounding a participant [15]. These include social contracts, physical spaces and societal norms [12]. Using an adapted Socio-Ecological Model [12], these factors can be separated into three broad categories: Individual level factors (i.e., intrapersonal factors), environmental factors (i.e., interpersonal, community and policy factors) and intervention factors (i.e., institution). *Individual level* factors include a participant’s personal motivation underlying their behavior change such as their knowledge, skills, attitudes, or perceptions about change. The *environment level* includes aspects of the physical environment (i.e., infrastructure) and systems within it (i.e., social influences or norms). Finally, the third category (i.e., *intervention level)* pertain to aspects of an intervention itself (i.e., its components, delivery, and location). Though broader factors, such as the “policy level” also play a role, they are often viewed as outside of an intervention or an individual’s control [12]. For this reason, focusing on more controllable aspects of an intervention and individual motivation can help interventionists make more actionable decisions about intervention design to improve participant adherence [15].

Understanding participant perceived facilitators or barriers to diet or activity interventions can inform their design (i.e., what) and delivery (i.e., how). More efficacious interventions in turn may facilitate greater uptake and maintenance of health protective behaviors [15]. Thus, the purpose of this systematic review is to explore participant perceived barriers and facilitators to diet and/or activity changes in lifestyle interventions at the individual, environment and intervention levels.

## Methods

This systematic review was registered and can be accessed at PROSPERO (ID: CRD42021262918). It has been conducted in accordance with the PRISMA Statement [25].

### Search strategy and selection criteria

A literature search was conducted in five databases (i.e., MEDLINE Ovid, PubMed, Web of Science, the Cochrane Library and Google Scholar). Google scholar was searched informally to help identify additional literature. Qualitative studies describing participant’s barriers and facilitators to adherence in diet and/or PA interventions were identified. Truncation and appropriate Boolean operators were used (Table 1). Since some interventions seek to change diet or activity habits for weight loss, we included the keyword ‘weight loss’ in the search syntax to expand the results. This way, an intervention flagged for ‘weight loss’ that aimed to change diet and/or activity behaviors would be captured. The databases were searched between January 2005 to October 2021. This period was chosen as the prevalence of adults living with overweight and obesity has been relatively stable over this time.

**Table 1:** Search syntax used in PubMed, Goggle Scholar, MEDLINE Ovid, Web of Science Core Collection, and Cochrane Library

Database	Outcome	Intervention			Study design	Dates	Population
PubMed	Barriers or facilitators or adher^a^ or prevent or motiv^a^	Diet or nutrition	Exercise or physical activity	Weight loss	Qualitative	"2005/01/01"[Date—Entry]: "3000"[Date—Entry]	Humans, English, Adult: 19 + years
Google Scholar^b^	Barriers or facilitators or adherence or prevent or motivation	Diet or nutrition	Exercise or physical activity	Weight loss	Qualitative	year 2005–2021	
Medline (OVID)	Barriers or facilitators or adher^a^ or prevent or motiv^a^ or motivation/	Nutrition or diet/ or diet^a^	Exercise/ or exercise or physical activity	Weight loss/ or weight loss	Qualitative research/ or qualitative	2005 to current	Human, English, all adults (19 plus years)
Web of Science	Barriers or facilitators or adher^a^ or prevent or motiv^a^	Nutrition or diet^a^	Exercise or physical activity	Weight loss	Qualitative	(2005–2021)	English, articles or Review articles
Cochrane Library	Barriers or facilitators or prevent or adher^a^ or prevent or motiv^a^	Diet or nutrition	Exercise or physical activity	Weight loss	Qualitative		

All search components (i.e., outcomes, intervention and study design) were combined using ‘and’. Dates and population components were added in as limits

^a^Truncates term to include all words with alternative endings

^b^Google scholar was searched informally

### Inclusion and exclusion criteria

Qualitative studies (e.g., interviews, focus groups or open responses writing data) that reported on participants perceived barriers or facilitators to behavior change during a lifestyle intervention were eligible. Lifestyle interventions were defined as those focused on changing diet (improving eating behaviors, diet quality) and/or activity (increasing frequency, type and duration) behaviors. Behavior change was defined in terms of participant perceived adherence to changing their diet and/or activity behaviors. In this way we considered ‘successes’ from the individuals’ perspective in their own words. All studies were conducted among adults (18–65 years). We chose to focus on adult populations as youth are often still confined to parental control over their behaviours, creating a situation where adherence is reliant on a third party [26]. Studies that included participants older than 65 years were eligible if the mean reported age was below 65 years.

Studies were excluded if they were conducted in populations living with dementia, cognitive impairment, physical disability, arthritis, HIV or undergoing cancer treatment. This approach was taken to control for underlying pain-related medication use that can impact diet or activity behaviors (e.g., pain from arthritis preventing activity). Studies conducted in pregnant women were also excluded. Letters, editorials, Masters and Doctoral theses were excluded. Systematic reviews of qualitative studies were back checked by hand for potentially relevant studies.

### Review section and data extraction

**Fig. 1:**
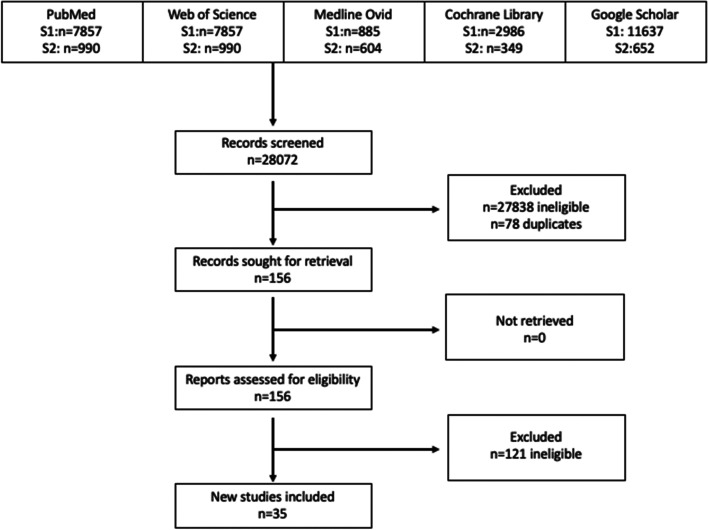
Flow diagram of included papers for review (*n* = 35)

The primary outcomes were perceived barriers and facilitators to participants’ adherence to diet and/or activity interventions. Two separate searches were conducted. In the first search (January 2005 to March 2020), three researchers removed duplicates and screened study abstracts divided by database (HB, MS, TC). One researcher then screened the full texts (MS) to determine eligibility in consultation with a second researcher when uncertainty existed (TC). This same researcher performed data extraction (MS). In the second search (April 2020 to October 2021), one researcher searched all data bases, removed duplicates and screened study abstracts (AD). Full text review was then conducted by the same researcher in consultation with a second researcher (AS). The second researcher then preformed all data extraction (AS). The second search was done to account for disruptions in the original study timeline caused by COVID-19. Figure 1 illustrates the flow chart for the study selection. Extracted details included study design, population (i.e., age, sex, ethnic or weight eligibility criteria), aim(s), methods, and findings (i.e., barriers and facilitators) and can be viewed in Table 2.

**Table 2:** Extracted information from all included studies (*n* = 35)

Study	Participants	Aim	Study design	Key themes	CASP score^a^
**Diet interventions**					
Abbott et al. 2010 [27] Australia	*n* = 23 adults (83% female) aged 19 to 72 years (mean 48.0 years) who self-identified as aboriginal	To explore the experiences of Aboriginal peoples who had attended cooking classes	Individual semi-structured interviews	Facilitators: New chronic health diagnosis, improve future health, family, be a role model for family, culturally appropriate foods, group learning, credible diet teachers Barriers: Food cravings, poor oral health, depression, moving around/travel, caring for others, cooking for others, social isolation caused by eating certain foods, lack of family support (“sabotage”), cost of healthier food	**6.5**
Astbury et al. 2020 [28] United Kingdom	*n* = 12 adults (75% female) aged 40–75 (mean 56.1 years) living in a larger body	To determine the clinical effectiveness, feasibility and acceptability of referral to a commercial low-energy total diet replacement program compared with usual weight management interventions in primary care	Individual semi-structured interviews	Facilitators: Health professionals, interventionist, feeling accountable to the research team, one-on-one dietary counselling, support for transition back to solid foods Barriers: Eating with other people, holidays, cooking for others, restriction of solid foods	**8.5**
Burke et al. 2009 [29] United States	*n* = 15 adults (80% female) aged 38–56 years (mean 48 years)	To explore participants’ reflections on their feelings, attitudes, and behaviors while using a paper diary to self-monitor their diet	Individual semi-structured interviews	Facilitators: Weight loss, gaining dietary knowledge, optimism towards changes, interest, commitment to intervention goals, other participants, accountability towards the research team, meal planning, self-monitoring, group learning, forming habits Barriers: Just didn’t want to, emotional eating, food cravings, fatigue, lack of time, housework, work, not interested or fun, forgetting, feeling overwhelmed by changes being asked	**6.5**
Dasgupta et al. 2014 [30] Canada	*n* = 29 adults (83% female) > 18 years (mean 58.9 years) living with type 2 diabetes	To explore effective elements of the strategy from participants’ perspectives after attending a nutrition education/meal training program	Focus groups	Facilitators: Weight loss, blood sugar control, gaining dietary knowledge, increased self-efficacy, reduced medicine intake, other participants, friends, being a role model for family, interactive programs, grocery store tours, incorporation of technology, self-monitoring, interactions with dietitians or chefs, monitoring activity with a pedometer	**7.5**
Hammarstrom et al. 2014 [31] Sweden	*n* = 12 women aged 49–71 years (mean 60 years) living in a larger body	To explore barriers and facilitators to weight-loss experienced by participants in a diet intervention	Individual structured interviews	Facilitators: Weight loss, commitment to intervention goals, other participants, family, friends, health professionals, interventionists, clear intervention goals/guidelines Barriers: Just didn’t do it, emotional eating, food cravings, lack of time, health issues (injury, dietary restrictions), travel/moving around, injury, stress with life changes, lack of motivation, previous failure managing health behaviors, coffee dates with friends, cooking with other people, lack of friend support, co-workers, family (“sabotage”), cost of healthier food, food accessibility, no health coach, transport to program locations, lack of food variety, no group cooking sessions	**7.5**
Maston et al. 2021 [32] Australia	*n* = 20 adults (60% female, mean age 51.2 years) living in a larger body	To explore facilitators and barriers to dietary adherence and program attrition in a specialized weight loss clinic	Individual semi-structured interviews	Facilitators: Weight loss, gaining dietary knowledge, other participants, health professionals/ interventionists, clear intervention goals/guidelines, stigma free spaces, self-monitoring Barriers: Emotional eating, unrealistic weight loss expectations, transportation to program, lack of food variety	**9**
Mendonca et al. 2019 [33] Brazil	*n* = 45 adults (58% female) $\ge$ 20 years (84.5% $\ge$ 40 years)	To investigate the barriers and facilitators for the adherence of participants in a 7-month nutritional intervention for promoting the consumption of fruits and vegetables	Individual semi-structured interviews	Facilitators: Gaining dietary knowledge, increased self-efficacy, interest, improve future health, commitment to intervention goals, other participants, family, health professionals/ interventionists, interactive design, clear intervention goals/ guidelines, nearby program locations, habit formation, length of meetings Barriers: Lack of time, housework, work, no weight loss, caring for others	**8.5**
Metzgar et al. 2015 [34] United States	*n* = 23 women (mean age 38.8 years) living in a larger body	To explore facilitators and barriers to weight loss and weight loss maintenance in women who participated in a primary, 18-week comparative trial that promoted weight loss with an energy-restricted diet	Focus groups	Facilitators: Gaining dietary knowledge, increased self-efficacy, having a routine for activity, feeling ready to change, other participants, family, friends, co-workers, health experts/interventionists, feeling accountable to others, flexibility in food choices, meal planning, group meetings, trusting interventionists, habit formation, monitoring by experts (dietitians), learning about portion control, weekly sessions Barriers: Food cravings, not losing weight, lack of motivation, feeling guilty taking time for oneself, lack of support from friends, co-workers, family (“sabotage’), feeling reliant on the program, no activity information/ components	**8.5**
Rehackova et al. 2021 [35] United Kingdom	*n* = 34 adults (53% female) aged 20–65 years (mean 51.0 years) living in a larger body with type 2 diabetes	To understand participant experiences in a clinical weight loss program relying on diet replacement and behavioral support	Individual semi-structured interviews	Facilitators: Weight loss, blood sugar control, increased energy, increased self-efficacy, motivated to change, other participants, modelling for others, feeling accountable to the research team, learning about hydration, trust in the diet information given Barriers: Hunger, fatigue, no weight loss, lack of food variety	**6**
Rodriguez, et al., 2020 [36] Mexico	*n* = 10 women aged 30–60 years (mean 47.6 years) with a higher prevalence and risk of living in a larger body	To provide a framework for the motivations of women to engage in and maintain a participation in a physician- dietitian nutritional counselling program	Individual interviews	Facilitators: Motivation change, family, friends, health professionals/ interventionists, wanting to look good, avoid health complications, weight loss, type of platform, gaining knowledge, group sessions Barriers: Food cravings, cost of programs and food, work, eating in social gatherings	**5.5**
**Activity interventions**					
Camhi et al. 2021 [37] United Stated	*n* = 26 women aged 21–78 years (mean 42.5 years)	To explore individual, interpersonal and environmental barriers and facilitators associated with participants adherence to an exercise prescription (ExRx)	Individual semi-structured interview	Facilitators: Feeling ready to change, family, friends, easy access to program location Barriers: Caring for others, program not meeting expectations, cost, no instructor for activity	**6.5**
Casey et al. 2010 [38] Canada	*n* = 16 adults (44% female) aged 39–65 years (mean 52.5 years) living with type 2 diabetes	To assess barriers and facilitators of participation in a supervised exercise program, and adherence to exercise after program completion	Focus groups	Facilitators: Stop medication, control blood sugar levels, improve physical well-being, improve mental wellbeing, weight loss, intervention staff, feeling accountable to research team, weather, family, peers, incorporation of walking, interactions in programs with activity experts Barriers: Physical health limitations (e.g., knee pain), lacking motivation, childcare, work, poor weather, transpiration to program, losing trainer, locations for activity after the program ended, strict program times for activity, lack of variety in activity type	**8.5**
Cooke et al. 2018 [39] Canada	*n* = 30 adults (47% female) aged 35 years and older (half 50–64 years) living with type 2 diabetes and/or hypertension	To assess participants’ experiences and impressions of an exercise intervention, to understand the perceived barriers and facilitators	Individual semi-structured interview	Facilitators: Improved physical well-being, intervention staff, family, friends, incorporation of walking, wearing a pedometer, doing activity daily, keeping an exercise diary, easy to understand guidelines/ content Barriers: Physical health limitations, work, poor weather, pedometer issues, loosing intervention support	**7.5**
Gallegos-Carrillo et al. 2020 [40] Mexico	*n* = 117 adults (68% female) aged 35–70 years (mean 50.4 years) diagnosed with high blood pressure	To explore the factors underlying adherence to a specific 16 weeklong exercise referral scheme (including group exercise sessions) aimed at increasing PA in hypertensive patients	Individual semi-structured interviews	Facilitators: More severe high blood pressure diagnosis, increased self-efficacy, prioritizing activity, less perceived barriers, family, friends Barriers: Concern for blood pressure when exercise, time, work, unwilling to preform activity, cost, lack of family support, transportation to program	**5.5**
McCormack et al. 2019 [41] Canada	*n* = 23 adults (83% female) aged 24 to 68 years (mean 37.5 years)	To explore individual, social, and physical environment characteristics that hinder or facilitate physical activity among previously “inactive” adults during a 12-week community-based internet-facilitated pedometer intervention	Individual semi-structured interviews	Facilitators: Committed to intervention goals, increased self-efficacy, decreased perceived barriers to activity, gaining knowledge, access to nature, companionship for activity, feeling accountable to research team, Family and friends, family, neighbourhood walkability, active transport, wearing a pedometer, goal setting, form habits Barriers: Poor weather and limited access to grocery stores, and lack of access to green spaces or nature	**8.5**
Eynon et al. 2018 [42] United Kingdom	*n* = 9 adults (56% female) aged 41–67 years (mean 49.9 years)	To identify the key psychological factors associated with adherence to an exercise referral scheme	Individual semi-structured interview	Facilitators: Committed to intervention goals, improve future health, increased self-efficacy, self-esteem, body image, prioritizing and enjoying activity, seeing physical improvements in activity abilities, emotional balance, forming an exercise identity, goal setting, monitoring, form habits	**8**
Huberty et al. 2008 [43] United States	*n* = 24 women aged 26 to 66 years (mean 46 years)	To qualitatively examine factors related to physical activity adherence to understand why women continue to participate in long-term exercise after completing a structured exercise program (“U Try Active Habits and Fitness” intervention)	Focus groups	Facilitators: Future health benefits, increased self-efficacy, self-esteem, self-worth, improved body image, prioritizing activity, enjoyment, feeling improvements in activity abilities, companionship for activity, intervention staff, family, friends, goal setting, continued interactions with activity experts, form habits Barriers: Time, lack of motivation, family care, self-conscious about body image when exercising, feeling guilty taking time to exercise, not enjoyable, fear of not achieving goals, low self-worth, lack of support from family, no one to be active with, opposing norms related to body size	**9**
Kinnafick et al. 2018 [44] United Kingdom	*n* = 12 adults (66.7% female) with a mean age of 39.7 years	To qualitatively evaluate the participant experiences of a workplace high-intensity interval training (HIIT) intervention targeting insufficiently active adults	Focus groups	Facilitators: Increased self-esteem, improved physical well-being, feeling improvements in activity abilities, enjoyment, feeling a sense of accomplishment, other participants, interventionists, neighbourhood walkability, flexible activity sessions times, being with a group with a similar activity level Barriers: Lack of time, belief that HITT is for ‘fit individuals,’ programs expectations not being met, no weight loss, poor weather	**7.5**
Korkiakangas et al. 2011 [45] Finland	*n* = 54 adults (55% female, mean age of 49 years) living with type 2 diabetes	To describe the motivators and barriers to physical activity among individuals with high risk of type 2 diabetes who participated in video group counselling sessions	Questionnaires with open-ended questions	Facilitators: Improved physical and mental well-being, weight loss, feeling improvements in activity abilities, enjoyment, forming an exercise identity, access to nature, companionship for activity, work environment, neighbourhood walkability, modeling for others, access to activity equipment, owning a pet Barriers: Physical health limitations, time, too tired, stress, not enjoyable, poor weather, season,	**6.5**
Lee et al. 2015 [46] South Korea	*n* = 27 women aged 36–60 years (mean 52.5 years). Migrant workers	To investigate the barriers to performing stretching exercise during a community-based 12-week stretching exercise intervention trial	Individual semi-structured interviews	Barriers: Health limitations (e.g., illness) lacking motivation, lack of time, family care, too tired, feeling incompetent preforming activity, lack of family support, co-worker support, no one to exercise with, travel	**5.5**
O’Dougherty et al. 2008 [47] United States	*n* = 80 women aged 25–44 years living in a larger body	To examine factors associated with adherence to a strength training intervention in a randomized controlled intervention trial	Focus groups	Facilitator: Committed to intervention goals, increased self-efficacy, improved body image, enjoyment, feeling a sense of accomplishment, accountability to others, family, friends, group activity sessions Barriers: Changes in schedule (i.e., moving or travel), time, family care, too tired, feeling overwhelmed to make changes, no weight loss, prioritizing social activities that are sedentary first, lack of family or friend support, no one to exercise with, opposing norms on physical appearance (e.g., women as muscular), no longer having group sessions, loosing activity trainer	**8.5**
Tulloch et al. 2013 [48] Canada	*n* = 28 adults (29% female) aged 39–70 years (mean 55.7 years) living with type 2 diabetes	To determine the perceived facilitators and barriers to exercise at multiple time points of participants in a randomized exercise trial including aerobic, resistance or combined exercise	Interviews	Facilitators: Future health benefits, control blood sugar, improve mental well-being, weight loss, improvements in activity ability, enjoyment, interventionists, family, interactions with activity experts Barriers: Time, family care, work, too tired, vacations, no interest/ not enjoyable, injury, illness, poor weather	**8**
Vetrovsky et al. 2019 [49] Czech Republic	*n* = 10 adults (30% female) aged 30–64 years (mean 43.7 years)	To explore patients’ experiences during a 12-week pedometer-based physical activity intervention in a primary care setting targeting inactive adults	Participant written email messages	Facilitators: Future health benefits, enjoyment, interventionists, family, owning a pet, incorporation of walking, wearing a pedometer, goal setting, self-monitoring, self-reward, forming habits Barriers: Lack of motivation, time, family care, feeling overwhelmed at changes, poor weather	**6.5**
Viljoen et al. 2015 [50] South Africa	*n* = 35 postmenopausal women aged 50 to 75 years (mean 58.9 years)	To assess self-reported motivational factors influencing adherence and retention to a resistance training program targeting sedentary women	Focus groups	Facilitators: Get off medication, increased self-worth, improved physical and mental well-being, weight loss, enjoyment, increased knowledge, social support, group activity sessions, flexible timing of activity sessions, form habits Barriers: Vacations, lacking social support	**7**
**Mixed interventions (diet and activity)**					
Brandt et al. 2018 [51] Denmark	*n* = 10 adults (70% female) aged 34–71 years (mean 52 years) living in larger bodies	To identify drivers of importance for long-term personal lifestyle changes from a patient perspective when using a collaborative e-health tool	Individual semi-structured interviews	Facilitators: Positive health changes, weight loss, healthcare/interventionists, family, friend, regular monitoring and communication with a healthcare expert, goals setting, trust in healthcare experts and the information they give Barriers: Lacking self-efficacy, feeling overwhelmed by changes, food cravings, illness, lacking family support, other participants, not feeling connected to interventionists, loosing monitoring by interventionists post program, not trusting information interventionists portray	**9**
Chan et al. 2009 [52] China	*n* = 25 adults (84% female) aged 18–62 years (60% $\ge$ 40 years) living in a larger body	To examine the participants’ experience of a community-based lifestyle modification program	Individual semi-structured interviews	Facilitators: Increased self-efficacy, gaining knowledge, self-control, positive health changes, weight loss, feeling full, healthcare professionals/ interventionists, family, clear guidelines, trust in healthcare experts and information given Barriers: Lack of motivation, self-efficacy, stress, work, time, fear of failing, lack of support from family, friends, colleagues, opposing usual social norms over behaviors, eating out or in social settings, short one-on-one sessions, lacking trust in interventionists or the information given	**8**
Hardcastle et al. 2011 [53] United States	*n* = 14 adults (64% female, mean age 57.6 years) living in a larger body	To explore the experiences of patients, following participation in a counselling intervention, to identify the influences on behavior change in relation to physical activity and diet	Individual semi-structured interviews	Facilitators: Increased self-efficacy, self-control, fear of negative health outcomes if don’t change, weight loss, commitment to intervention goals, social support, healthcare experts/ interventionists, feeling accountable to others, regular monitoring and communication with health experts, pedometers, interactions with diet or activity experts, form habits, planning ahead Barriers: No weight loss, lack of support from family (“sabotage”), no connection to interventionists, no one to exercise with, rigid intervention structure/delivery times, support for behavior change, just information on what to change	**9**
Harrison et al., 2020 [54] United States	*n* = 29 women aged 18–39 years (mean 30.3 years) living with type II diabetes	To understand barriers and facilitators to engagement in a national Diabetes Prevention Program (year-long, group-based program with lifestyle coaches) for young women at an urban safety-net health care system	Individual semi-structured interviews	Facilitators: Motivated to change lifestyle, avoid chronic disease development, weight loss, healthcare experts/ interventionists, support from other participants Barriers: Work, unclear guidelines, onsite weighting (stigma), transportation to program locations, wide range of age groups in sessions together	**7.5**
Kleine et al. 2019 [55] United States	*n* = 61 adults (70% female) aged 31 to 82 years (mean age of 51 and 56 years in two separate cohorts	To identify barriers and facilitators of weight loss and weight loss maintenance among individuals participating in a meal replacement program from lower socio-economic areas	Focus groups	Facilitators: Increased self-efficacy, gaining knowledge, feeling accomplishment, positive changes to health, weight loss, changes in body shape, increased energy, commitment to intervention goals, improved quality of life, increased mobility, support from healthcare experts/ interventionists, family, other participants, clear guidelines, educational hand-outs, form habits, quick choices (food) that can be taken on the go, interactions with diet/ activity expert Barriers: Time, lack of family support, not feeling connected to interventionists, eating out or in social settings, cooking for others, loosing monitoring with others post program, being sick of dietary replacement foods, not talking about activity with a health coach, lacking trust in interventionists or the information they provide	**7.5**
Leung et al. 2020 [56] China	*n* = 26 adults (79% female) aged 18–65 years (mean 38.9 years) living in a larger body	To explore the psychological factors of dietary and physical activity adherence 10 months after enrolment in a community-based lifestyle modification program	Individual semi-structured interviews	Facilitators: Increased self-efficacy, gaining knowledge, positive changes to health, weight loss, changes in body shape, healthcare experts/ interventionists, family, friends, monitoring heart rate during activity, interactions with diet or activity experts, paying for the program, form habits Barriers: Forgetting what was taught, no one to exercise with	**9**
Lieffers et al. 2020 [57] Canada	*n* = 32 first year university students (72% female) aged 17–30 years	To understand experiences and perception of web-based application that aims to support healthy living by providing resources and self-monitoring tools in a12-week randomized controlled trial	Individual semi-structured interviews	Facilitators: Gaining knowledge, feeling accomplishment, daily reflection, goal setting Barriers: Feeling overwhelmed by changes, cost, rigid intervention structure, lack of culturally inclusive food, no app	**8.5**
Penn et al. 2013 [58] United Kingdom	*n* = 15 adults (53% female) aged 40– 65 years (mean 54 years) at elevated risk of type 2 diabetes	To explore participants’ perspectives of their behavioral change and maintenance of new behaviors in physical activity intervention	Individual semi-structured interviews	Facilitators: Increased self-efficacy, gaining knowledge, motivated to change lifestyle, feeling accomplished, avoiding chronic disease development, control diabetes, weight loss, improved body image, feeling good after exercise, friends, health experts/ interventionists, free gym access, filling free time caused by retirement (enjoyment) Barriers: No weight loss, embarrassed performing activity, not wanting to fail a set goal, cost	**6.5**
Rise et al. 2013 [59] Norway	*n* = 23 adults (61% female) aged 35–72 (mean 58 years) living with type 2 diabetes	To investigate how participants make and maintain lifestyle changes after participating in group-based type 2 diabetes self-management education	Individual semi-structured interviews	Facilitators: Increased self-efficacy, gaining knowledge, fear health complications if don’t change, diabetes control, positive changes to health, weight loss, increased energy, support from healthcare experts/ interventionists, family, friends, educational handouts, forming habits Barriers: Time, lack of support from family	**8.5**
Schmidt et al., 2020 [60] Norway	*n *= 6 adults (50% female) aged 41–74 years (mean 56.5 years) living with type II diabetes	To explore and identify factors that influence motivation for and barriers to adopting and maintaining lifestyle changes following participation in an intensive multiple-lifestyle intervention	Individual semi-structured interviews	Facilitators: Increased self-efficacy, decrease medication, improved fitness ability, support from healthcare experts/ interventionists or other participants, co-participation for activity Barriers: Stress, injury, work, opposing social norms over behaviors, rigid intervention structure, loosing monitoring support when intervention ended	**7.5**
Wycherley et al. 2012 [61] Australia	*n* = 30 adults (27% female, mean age 56.7 years) living with type 2 diabetes and a larger body	To document factors reported that enhanced or impeded sustainability of lifestyle behaviors following participation in a 16‐week lifestyle intervention program	Individual semi-structured interviews	Exercide barriers included: reduced access to gyms, equipment or similar exercise programmes (29%) and the expense of public gyms (21%) as impediments Facilitators: Increased self-efficacy, gaining knowledge, feeling accomplished, diabetes control, positive changes to health, weight loss, support from healthcare experts/ interventionists, family, feeling accountable to research team, regular monitoring with interventionists, monitoring heart rate during activity, activity encouraging dietary maintenance, clear guidelines, interactions with experts, portion control, forming habits Barriers: Feeling overwhelmed by changes, start activity, food cravings, travel, cost, lack of access to gyms, rigid intervention structure, restriction of alcohol, loosing monitoring support after an intervention ended, no activity discussion with a health coach	**7**

*n* = number. ^a^CASP score: Quality assessment measure scored out of 10 points

### Data analysis

Two researchers created the code book using inductive coding after reviewing all extracted themes from studies identified in search one (AD, CB). To do this, extracted themes from studies in search one were separated into three broad categories borrowed from SEM (i.e., individual, environment and intervention levels) [12]. Then, inductive codes borrowing language from published work were used to name sub-themes with the personal, community and intervention spheres. This included using terminology from behavior change taxonomies (BCTs) [62], behavior change theories (e.g., motivation, knowledge, attitudes) [11] and definitions of *what* and *how* components of interventions [20]. Sub-theme names were not deductively applied, but instead used to guide final decisions as all themes inductively emerged from the extracted data itself. Guidelines do not currently exist on how to consolidate emergent themes across studies. As a result we opted to rooted our analysis in behavioural science terminology as other work has suggested that this approach can help facilitate relevant understanding and application within the field of behavioural science [63]. After the codebook was established, it was independently applied by two researchers to all studies identified in search two (AD, AS). Discrepancies in theme names or coding of studies was triangulated with a third researcher (CB).

### Quality assessment

The methodological quality of all papers was assessed using the Critical Appraisal Skills Programme (CASP) checklist for qualitative research [64, 65]. This appraisal tool consists of ten questions. In the appraisal, responses to each question were checked off as ‘yes’, ‘no’ or ‘can't tell.’ Afterwards, a ‘somehow’ group was added. An overall quality score for each article was then assigned as follows: 1. ‘Yes’ assigned one point; 2. ‘Somewhat’ assigned a half point; and 3. ‘No’ or ‘can’t tell’ assigned zero points for each of the 10 questions. The maximum achievable score was 10 points [64]. The methodological quality of all included studies was evaluated by teams of two researchers in each of the two searches (AD, AS). Discrepancies were discussed until consensus.

## Results

Titles and abstracts were reviewed for 28,072 papers (25,162 search one, 2,910 search two). Seventy-eight duplicates (54 search one, 24 from search two) were removed. After screening, 156 papers were identified for full text review (129 search one, 27 search two) (Fig. 1). The eligibility criteria were applied resulting in a final sample of 35 papers (24 search one, 11 search two). Ten of these looked exclusively at diet-focused interventions [27–36]. Of these, one reported only on facilitators [30]. Fourteen studies exclusively at activity behaviors [37–50] with one reporting only facilitators [42] and one only barriers [46]. Eleven studies reported barriers and facilitators in mixed interventions [51–61].

### Study designs

Semi-structured interviews (*n* = 22) [27–29, 32, 33, 35, 37, 39–42, 46, 51–54, 56–61], structured interviews (*n* = 1) [31], unspecified interviews (*n* = 2) [36, 48], focus groups (*n* = 8) [30, 34, 38, 43, 44, 47, 50, 55] and open written responses (*n* = 2) [45, 49] were used.

### Study populations

Ages of included participants varied from 17 to 82 with an overall mean age of 49.7 years (diet: 50.9 years, activity: 49.1 years and mixed: 49.9 years) in 30 studies. Five studies did not present data to calculate a mean age [33, 39, 47, 52, 57]. Forty-six percent of studies were conducted in North America, four diet [29, 30, 34, 36], eight activity [37–41, 43, 47, 48] and four mixed [53–55, 57]; 31% in Europe, three diet [28, 31, 35], four activity [42, 44, 45, 49] and four mixed [51, 58–60]; 9% Australia, two diet focused [27, 32] and one mixed [61]; 9% East Asia, one activity [46] and two mixed focused [52, 56]; 3% Africa, one activity focused study [50] and 3% South America, one diet focused [33]. Seventy-four percent of studies (*n* = 26) included both males and females [27–30, 32, 33, 35, 38–42, 44, 45, 48, 49, 51–53, 55–61]. Of these, only 14% (*n* = 5) had more male participants than female and none of these were diet focused interventions. Twenty-six percent (*n* = 9) of studies recruited only females [31, 34, 36, 37, 43, 46, 47, 50, 54]. Sample sizes ranged from 6 to 117 participants (mean: 28.1). On average, ages in activity interventions were higher (diet: 22.3 years, activity: 35.1 years, and mixed: 24.6 years).

### Review quality

The CASP scores ranged from 5.5 to 9.5. This indicates that included studies were primarily of moderate to high quality [64, 65]. The lowest scoring domain was recruitment. Many studies did not describe participant characteristics and only one study included details on saturation. Several studies also failed to include information on researcher-participant relationships.

### Primary findings

Facilitators and barriers that arose across all three level were often an inverse of each other. For example, having a positive attitude was a facilitator whereas having a negative attitude was a barrier. At the individual level, three themes (attitudes, concern for health and physical changes) emerged. Four more at the environment level (social support, social accountability, changeable aspects of the community and unchangeable aspects of the community) and two at the intervention level (delivery and design and content) also arose. One additional theme called ‘fostering self-regulation through BCTs’ emerged as a facilitator only at the intervention level. A description of the emergent themes and their sub-themes from all studies can be found in Table 3. The remaining sections will discuss how themes differed across different intervention types (diet, activity and mixed).

**Table 3:** Emerging facilitators and barriers participants felt impacted their ability to adhere to intervention guidelines for diet and/or activity behaviors

Level	Theme	Facilitators		Barriers	
		Definition	Main codes (tertiary codes)	Definition	Main codes (tertiary codes)
Individual	Attitudes	Expressing a positive opinion of the intervention or behavioral guidelines within it	Desire for knowledge, positive initial mindset, changes in self-perception, Paid for it	Expressing a negative opinion of the intervention or behavioral guidelines within it	Negative self-perception, competing priorities (work outside the home, work inside the home, life events, inner food cues), feeling over-whelmed, unwilling to change
	Concern for health	Intervention provided a means for a participant to take control of their health for the better	Current health, Avoiding future diagnosis	Concern for or presence of physical and mental complications to changing behaviors	Physical state, Feeling low
	Physical changes	Positive view of physical changes or abilities with intervention adherence	Body shape, brain-body connections (vitality, satiety)	Over emphasis or expectations on changes in body shape	Focus on weight
Environment	Social support	Physical, practical and emotional support from others for behavioral change or maintenance	Within the intervention, within the home, outside of the home (at work, peers)	Lack of physical, practical and emotional support from others for behavioral change or maintenance	Within the intervention, within the home, outside of the home (at work, peers)
	Social accountability	Maintaining behavioral adherence based on internalized pressure or accountability towards other people	Participation guilt, being a role model, changing for others	Difficulty overcoming traditional norms or expectations on diet and/or PA activities around other people	Opposing social norms
	Changeable community aspects	Presence of infrastructure and physical spaces with the community that support adherence and can be targeted through interventions or policy	Built environment	Lacking infrastructure or physical spaces with the community that facilitate practice of new diet or activity behaviors that can be targeted by interventions or policy	Built environment, cost,
	Unchangeable community aspects	Factors unique to a community that are not changeable by individuals that facilitate adherence	Weather	Factors unique to a community that are not changeable by individuals that hinder adherence	Weather
Intervention	Delivery and design	Positive opinions on the methods, physical spaces and delivery platforms used within an intervention that fostered adherence	Nearby location, inclusive spaces, flexible delivery routes, opportunities for social support (with peers, with professionals), support after the intervention period	Negative opinions on the methods, physical spaces and delivery platforms used within an intervention that hindered adherence	Far away sites, opportunities for stigma, one size fits all, limited social engagement (with participants), Intervention reliance
	Content	Opinions on aspects of intervention information and how it was portrayed that supported diet or activity adherence	Perceived credibility, lifestyle management, clarity in messaging, tailoring	Opinions on aspects of intervention information and how it was portrayed that hindered diet or activity adherence	Distrust, lack of tailoring, lack of activity information, unclear messaging
	Fostering self-regulation (BCT)	Active ingredients within the intervention that fostered engagement and gain of self-regulatory skills	Feedback and monitoring (self-monitoring, professional monitoring), demonstrations, forming habits, tangible rewards, goals and planning (goal setting, planning ahead)		

*BCT* Behavior change taxonomies

### Individual level

Individual attitudes played a large role in motivating behavior adherence. Participants talked about how their ‘desire for knowledge [27, 29, 30, 32–34, 41, 50, 52, 55–59, 61] (e.g., interest in learning or gaining knowledge), ‘positive initial mindset’ [29, 31, 33–37, 40–45, 47, 47–50, 53–55, 58] (e.g., optimism for changes or commitment to intervention goals) or experiencing ‘changes in self-perception’ [30, 33–35, 38, 40–45, 47, 48, 50, 52, 55–58, 61] (e.g., increased self-efficacy or feeling pride with achieving goals) had benefits on their perception of the intervention or its guidelines, which fostered adherence. In one study, having a positive attitude towards the intervention was conferred due to the notion that it was ‘pa[id] for’ [56]. Participants also discussed how their positive attitude towards an intervention was influenced by different desires in diet interventions compared to activity interventions. For example, in diet interventions participants focused on changes in their physical skills and abilities through wanting to gain knowledge of how to eat better or learning new food-related skills (e.g., new healthy recipes) [27, 29]. This contrasted motivation of participants in activity interventions, where the focus surrounded wanting to change aspects of they viewed themselves (i.e., their self-perception through changes in self-esteem [50, 52, 61], self-worth [33, 61] or mood [33, 34, 50, 52, 54, 56]) or how others viewed them (i.e., by forming an exercise identity [50, 56]).

Having a negative attitude towards the intervention or its guidelines hindered adherence. Participants revealed that ‘negative self-perception’ [29, 31, 33, 38, 43–46, 49, 51, 52, 58] (lacking self-efficacy or motivation to change behaviors), too many ‘competing priorities’ [29, 31, 33, 34, 38, 40, 43–49, 52, 54–56, 59, 60] (e.g., lack of time), ‘feeling overwhelmed’ [29, 31, 43, 47, 49, 51, 52, 56–58, 61] (e.g., previous failure in changing behaviors or not knowing how to start) or being ‘unwilling to change’ [29, 40, 43, 45, 48] (e.g., not interested) negatively impacted their attitudes and likelihood of adherence. In interventions with a diet component, ‘inner food cues’ like hunger, food cravings or emotional eating arose as a unique competing priority [27, 29, 31, 32, 34, 35, 51, 56, 61]. No equivalent discussion of feeling uncontrollable urges to be active or inactive were discussed in activity interventions. Other competing priorities common across all intervention types included ‘work outside of the home’ [29, 33, 38, 40, 44, 48, 52, 54, 55, 60], ‘work inside the home’ [27, 29, 33, 34, 38, 43, 46–49, 55] (e.g., caring for others) or unforeseen life events [27, 31, 46–48, 50, 61] (e.g., moving or travel).

Concern for ‘current health’ [27, 30, 35, 38, 40, 48, 50–52, 55, 56, 58–61] (e.g., positive changes) and ‘avoiding future diagnosis’ [27, 33, 42, 43, 48, 49, 53, 54, 58, 59] (e.g., chronic disease development) facilitated adherence to diet and/or activity interventions. As a barrier, health concerns for ‘physical state’ [27, 29–31, 33, 38–40, 45, 46, 48, 50, 51, 60] (e.g., illness or injury) or ‘feeling low’ [27, 29, 35, 43, 45, 47, 48, 52, 56, 60] (e.g., fatigue, low mood or depression) prevented behavior changes. No differences between the different types of interventions emerged.

Observations of physical changes signalled to participants that an intervention was working. This helped reinforce commitment to continue to pursue behavior guidelines. Observed changes in ‘body shape’ [29, 30, 32, 35, 38, 42–45, 47, 48, 50–56, 56, 59, 61] (e.g., weight loss or body image) or ‘brain-body connections’ [35, 38–40, 42–45, 48, 50, 55, 58–60] (e.g., feeling stronger or having more energy) acted as facilitators. As a barrier, participants exclusively talked about how failing to see changes in weight or having a ‘focus on weight’ often led to dissatisfaction in progress, hindering behavior maintenance [32–35, 44, 47, 52, 53, 56, 58]. Though discussed in all interventions, those with an activity component were more often mentioned to contribute to changes in brain-body connections through increased perceptions of physical well-being or abilities to preform activities (e.g., greater mobility or abilities to perform activity) compared to diet (e.g., increased energy levels only).

### Environment level

Social support was the most frequently identified facilitator and barrier, talked about by participants in all interventions. This included support ‘within the intervention’ [28–36, 38, 39, 43, 44, 48, 49, 51–56, 58–61] (e.g., other participants, intervention staff and health experts like dietitians, trainers or doctors), ‘within the home’ [27, 31, 33, 34, 36–41, 43, 47–49, 51, 52, 55, 56, 59, 61] (e.g., family) and ‘outside of the home’ [30, 31, 34, 36–40, 43, 45, 47, 51, 56, 58, 59] (e.g., at work or with peers). In activity interventions having a pet or someone to by active with [41, 43, 45, 49] (e.g., co-participation) also supported behavior changes. No mention of having someone to preform dietary behaviors with like eating, cooking or grocery shopping was mentioned.

‘Social accountability’ also arose as a key influence. As a facilitator, feeling ‘participation guilt’ [28, 29, 35, 38, 39, 41, 44, 47, 50] (e.g., not wanting to let the research team down), wanting to ‘be a role model’ [27, 30, 35, 41, 45] or ‘change for others’ [34, 51, 53, 56, 58, 61] (e.g., feel accountable to change for family and friends) promoted diet or activity adherence. Contrasting this, ‘opposing norms’ or social contracts was talked about as a barrier [27, 28, 30, 31, 43, 47, 52, 55–57, 60]. In diet interventions opposing norms on the types or quantities of foods that are typically prepared (e.g., family or cultural norms), consumed with others (e.g., baked goods at coffee shops with friends) or a part of celebrations (e.g., holidays) were frequently discussed. Only two studies mentioned opposing norms in activity studies with both suggesting pressures to change to meet the typical convention surrounding body shape for women [43, 47].

‘Changeable’ (e.g., built environment and cost) and ‘unchangeable community aspects’ (e.g., weather) were mentioned mostly by participants regarding activity behaviors. As both a facilitator and a barrier, aspects of the built environment like access to nature [41, 45], nearby stores or recreation sites impacted activity practices [41, 44, 45]. No mention of infrastructure to cook or nearby food outlets was suggested by participants as a facilitator, but it did arise as a barrier [31, 57]. Cost was exclusively mentioned as a barrier in all types of interventions [27, 31, 37, 40, 57, 58, 61]. Finally, unchangeable aspects such as the weather to support outdoor activities, especially walking, was discussed only in activity studies [38, 39, 41, 44, 45, 48, 49].

### Intervention level

Having ‘nearby locations,’ [33, 37, 58] ‘inclusive spaces’ [32] (e.g., stigma free), ‘flexible delivery routes’ [30, 33, 44, 50, 55, 58, 59] (e.g., timing of counselling sessions or incorporated some on site and at home components), ‘opportunities for social support’ [27–30, 33, 34, 38, 43, 44, 47, 48, 50, 53, 55, 56, 58, 60, 61] (e.g., with peers and with professionals) and ‘support after the intervention period ends’ [43, 53, 58] (e.g., post program info or resources to help with the transition to doing things without interventionists) were suggested intervention facilitators. Their inverse including ‘far away sites’ [31, 32, 38, 40, 54], ‘opportunities for stigma’ [54] (e.g., onsite weighing), ‘one size fits all’ [38, 39, 52–54, 57, 60, 61] (e.g., rigid structure or timing of sessions), ‘limited social engagement’ [31, 47] (e.g., with participants) and ‘intervention reliance’ [29, 34, 38, 39, 47, 51, 55, 60, 61] (e.g., delivery that was solely reliant on intervention for monitoring or spaces to facilitate behavior uptake) were reflected as barriers. In interventions where participants viewed interventionists as ‘experts’ (e.g., fitness instructors, diet specialists, research team members), adherence was facilitated [28, 30, 34, 49, 51, 53, 56, 58, 61]. This was largely attributed to perceived knowledge and their trusting the interventionists [27, 34, 35, 38, 43, 48]. It was also influenced by interventionists having a recognizable title (e.g., dietitian) [30], or through interactions where the interventionists modelled or provided corrections to an individual’s actions in physical activity interventions, for example [53, 55, 56, 58, 61]. Conversely, when participants did not feel that interventionists were knowledgeable, they did not perceive them as experts and were less open to follow their advice [51, 52, 55]. Comments surrounding inclusive spaces and opportunities for stigma was mentioned in a single diet focused study [54]. All other themes were consistent across intervention types.

Intervention content was viewed as a facilitator when it was ‘perceived credible’ ​[27, 34, 35, 51, 52] (e.g., trust information provided or who delivered it), had ‘clarity in messaging’ [31–33, 39, 52, 55, 61] (e.g., clear guidelines or goals), and allowed for ‘tailoring’ [27, 34, 56, 58] (e.g., flexibility in food choices or activity type). In diet focused interventions, content that included ‘lifestyle management’ [30, 34, 35, 55, 61] such as information on physical activity was also suggested to support adherence. No mention of dietary information supporting adherence for activity was mentioned in any studies. As barriers, ‘distrust’ [32, 51, 52], ‘lack of tailoring’ [27, 28, 31, 32, 35, 38, 55, 57, 61], ‘unclear messaging’ [37, 44, 54, 55] and ‘lack of activity information’ [34, 55, 61] arose.

Fostering self-regulation through incorporation of different BCTs in interventions was perceived by participants to have positive impacts on their adherence. This included ‘feedback and monitoring’ through self-monitoring [29, 30, 32, 35, 39, 41, 42, 45, 49, 53, 57, 58] (e.g., using pedometers, diaries) and professional monitoring [28, 34, 49, 51, 53, 56, 58, 61] (e.g., counselling support or check-ins). ‘Goals and planning’ through goal setting [41–43, 49, 51, 57], planning ahead [29, 34, 40, 58]. Demonstrations [30, 56] through grocery store tours or trainer demonstrates for activity) and forming habits [29, 33, 34, 38, 39, 41–43, 45, 49, 53, 55, 56, 58, 59, 61] through habitual practice of activities like walking or meal planning. Use of ‘tangible rewards’ to self-reward arose in one activity study [49]. All other themes were consistently stated regardless of intervention types. Presence or absence of BCTs was not recognized as a barrier.

## Discussion

**Fig. 2:**
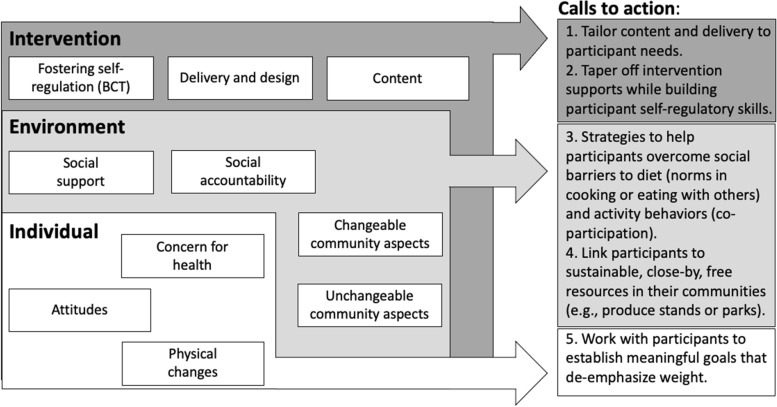
Calls to action for interventionists based on common facilitators and barriers in lifestyle interventions at the individual, environment and intervention level

We explored in a systematic review of 35 lifestyle (diet and/or activity) interventions participant perceived facilitators and barriers to behavior adherence at the individual, environment and intervention level. By consolidating these similarities and differences across intervention types, our findings add to the literature by suggesting actions that interventionists can implement now to help overcome common barriers. This has the potential to improve intervention design or implementation and in turn may increase participant adherence, leading to improvements in health and wellbeing. A summary of relevant themes and there call to action for intervention developers can be found in Fig. 2**.**

### Individual level

At the individual level, common facilitators for both diet and activity interventions centred predominately around psychological factors (desire for knowledge, positive mindset, self-perception), self-regulatory skills (overcoming barriers with perceived lack of time or feeling overwhelmed) and observations of physical changes (weight, physical health or sense of wellbeing). This supports findings from a 2015 systematic review suggesting that psychological or self-regulatory skills and body image act as effective mediators of behavior change in lifestyle interventions [66]. However, our findings also revealed that focusing on weight can hinder behavior changes when expectations are not met.

Unrealistic expectations surrounding weight changes can lead to discouragement [67]. It also prevents an understanding of the importance of health behaviors on internal changes like blood pressure. For example, in one study in this review the authors found that participants empathized how positive changes in their body composition or energy were not motivating unless other people acknowledged them [47]. Counter-acting a focus on weight may be one strategy to help prevent discouragement with a lack of outward physical changes in lifestyle interventions [47, 68]. Other interventions within this review support this approach as changes in energy [35, 38, 42, 44, 45, 48, 50, 55, 59], confidence [30, 33–35, 40–43, 47, 52, 55, 56, 58, 60, 61], physical abilities [40, 42, 43, 45, 48, 55, 58, 60] or general health [38, 39, 44, 45, 50] acted as facilitators. This creates a twofold call to action for interventionists. First, a greater understanding of participants personal goals prior to intervention onset and second, increasing participants’ understanding that changes beyond weight are valuable [47, 68]. This matches findings from a recent systematic review looking at barriers and facilitators in 13 community-based physical activity interventions. In this study, researchers concluded that strategies such as negotiated planning and fostering individual buy-in are critical for intervention success [15]. Furthermore, community engagement strategies like those used by Bryne (2019) may provide one approach to better understand participant goals of a target group from the start to help shape outcomes in intervention design phases [69].

The most common barrier across all interventions was competing priorities and time constraints [27, 29, 31–35, 38, 40, 43–51, 54–56, 59–61]. Though not surprising, supporting participants to feel like diet or activity changes can be adopted as a part of a regular routine may facilitate behavior change [70]. This matches suggestions from participants in this review discussing how components in interventions that encouraged habit formation [29, 33, 38, 39, 41–43, 45, 49, 53, 55, 56, 58, 61] and incorporated planning [29, 34, 40, 58] were beneficial. Interventions should focus on helping participants find ways to easily incorporate guidelines into their schedule as feasible habits. This can be achieved through aspects like tailoring content [27, 34, 56, 58] or flexible intervention delivery (i.e., location or timing) [30, 33, 50, 55, 58, 59]. Interventionists could also take an approach of helping participants predict barriers that they may face to pre-emptively have strategies in place when anticipated barriers arise [15].

### Environmental level

Participants in all interventions discussed the importance of social support from various entities. This is supported by other literature [71–73]. Interestingly, social support as a barrier looked different in diet compared to activity interventions. Lacking social support in dietary interventions was perceived to cause social isolation. For example, participants felt that they had to oppose typical norms surrounding eating in social settings like at the workplace, during holidays or social gatherings [28, 31, 52, 55, 56, 60] and when feeding other people [27, 28, 30, 31, 55, 57]. In contrast, in activity interventions, a lack of social support prevented action. For example, not having a companion for exercise [34, 43, 46, 47, 53, 56]. Based on these differences, targeted strategies based on targeted behavior type may be needed. Diet interventions could focus on breaking down discomfort opposing norms in eating with other people [74] or when cooking for others [75]. In activity interventions, social support strategies could utilize group activities [47, 50, 58, 60] or create opportunities for companionship (e.g., walking with other people or pets) [45, 49]. Helping participants identify solo activities that they enjoy is also likely important to prevent intervention reliance [76]. This could include strategies for indoor activities as well to avoid barriers suggested by participants regarding weather [38, 39, 41, 44, 45, 48, 49].

The built environment, including infrastructure, access to active spaces, healthy food and cost were common barriers across interventions [27, 31, 37, 40, 41, 44, 45, 57, 58, 61]. Though these aspects are difficult to address within interventions themselves, they are still noteworthy. Creating an ‘implementation plan’ may help reveal barriers at the community level that could hinder intervention implementation or adherence by participants [15, 77]. Context specific plans may help interventionists identify barriers and control for them in intervention design. For example, subsidizing or covering external costs associated with behaviour change like the cost of healthy food, gyms memberships or physical activity equipment. This has proven to be beneficial for compliance and consequently in improvement of intervention outcomes in low sodium diet [78–82] and activity interventions [83]. However, this type of approach can create intervention reliance [29, 34, 38, 39, 47, 51, 55, 60, 61] and should be evaluated for its potential to limit sustainable change once an intervention ends. Early identification of possible barriers outside of an individual’s control at the intervention level through an implementation plan may help shape design, helping overcome more systematic barriers from the start [15, 77].

### Intervention level

A common theme at the interventional level was a lack of support for participants once an intervention ended [29, 34, 38, 39, 47, 51, 55, 60, 61]. This could have been attributed to participants discussion of reliance on intervention tools or experts for monitoring [28, 30, 32, 34, 49, 51, 56, 58, 61], or losing accountability to interventionists [28, 29, 35, 38, 39, 41, 44, 50]. This creates an environment where external motivation fuels behaviors [76] and can be problematic. Many participants talked about wanting to have regular interactions with ‘experts’ like activity trainers or dietitians in interventions to guide behavior change [28, 30, 33, 34, 38, 43, 48, 53, 55, 56, 58, 61]. It is worth noting however, that participants included within this systematic review and by others [15] quickly point out that when ‘experts’ in an intervention are not perceive as skilled, these interactions act as a barrier [51, 52, 55]. To overcome this, it is key for trained, credible interventionists to guide participants through behavior changes while fostering development of self-regulatory skills [50, 61]. One option to support this is by tapering off intervention supports. It could also include providing resources that can be used long after an intervention period has ended [28, 43, 58]. This approach has been suggested regarding intervention implementation (i.e., stepwise implementation) in a similar vein [15]. Complementary delivery routes that include mobile health (mHealth) may help satisfy this need as they can continue to be used autonomously by participants after an intervention period [84–86]. Future work should explore if the inclusion of mHealth can supplement traditional in-person interventions to better support participants once an intervention has ended.

Participants talked about how activity helped facilitate dietary behavior change, but not the other way around. It is not clear why this arose. Some literature has suggested positive benefits for more holistic interventions that focus on multiple behaviors [87]. Therefore, there is a need to consider if combining behaviors, including exploration of other health behaviours like sleep, has benefits on long-term behavior change compared to interventions that target one behavioral realm.

Finally, key facilitators and barriers among all interventions surrounded a lack of personalization to unique needs, goals, interests and schedules. This suggests that tailoring of interventions can support incorporation of new diet or activity behaviors. Individualized interventions where participants have bought into an intervention [15] have been shown to be more effective [88–92]. Goal setting [93–95] or self-monitoring [96–98] may be examples of effective BCT to help personalize interventions while prompting self-regulation [93–95, 99], as these BCTs have shown promise as facilitators of intervention adherence [27, 30, 32, 37, 39, 41–43, 45, 49, 51, 53, 56–58, 61, 96]. However, these BCT have their own set of challenges [93, 100] and research is needed to understand when different BCT offer merit. To do this, we echo calls for clear identification and classification of BCT within interventions first to facilitate greater exploration as to when different BCTs work [14].

### Limitations

This review is not without limitations. First, the search strategy did not include targeted MeSH (medical subject heading) terms. This may have contributed to some studies being missed in each database depending on how they were indexed. However, it is more likely that a larger number of articles were returned and screened using this approach and instead, semi-related studies were reviewed (and excluded). The review protocol is also limited by having a single researcher lead full text review in consultation with a second researcher instead of having two independent researchers review all texts. Furthermore, caution needs to be present when interpreting these findings and making extrapolations to different sex, gender, age or cultural groups as the results above are largely representative of the opinions of females over the age of 40 years from North America. Many studies also ranked low in their quality of recruitment methods and did not outline if saturation was reached. This could imply that themes from studies with smaller samples in this review are not exhaustive. Lastly, though we rooted our analysis in a modified SEM (i.e., individual, environment and intervention levels), we did not acknowledge the nuances in how factors can act at multiple levels. For example, ‘cost’ can be rooted in preferences of what to spend money on (individual level), the cost of living (environment level) or failure of an intervention to provide certain supports (intervention level). In this paper, facilitators and barriers were viewed as mutually exclusive at one level, though they can be intertwined, which may have resulted in an oversimplification of the findings.

## Conclusion

Incorporating strategies to mitigate barriers participants face within lifestyle interventions at the personal, environment and intervention levels may help promote behavior adherence. This includes: 1. Understanding participant unique goals and de-emphasizing weight-related outcomes; 2. Providing opportunities for diverse social companionship; 3. Anticipating personal and intervention level barriers in advance of intervention onset; 4. Preventing intervention reliance by fostering self-regulatory skills (i.e., rooting in BCT); and 5. Tapering off intervention supports. Greater adherence to intervention guidelines may support the uptake and maintenance of new diet or activity habits, supporting lifelong health.

## Data Availability

Please contact the corresponding author for supplementary data and material.

## Abbreviations

BCT: Behavior Change Taxonomies
CASP: Critical Appraisal Skills Programme
mHealth: Mobile health
SEM: Socio-Ecological Model

## References

[1] National Center for Chronic Disease Prevention and Health Promotion. Physical Activity Prevents Chronic Disease [Internet]. CDC Centers for Disease Control and Prevention. 2020 [cited 2020 Dec 17]. Available from: https://www.cdc.gov/chronicdisease/resources/infographic/physical-activity.htm.

[2] Dietitians of Canada. Weight/Obesity Summary of Recommendations and Evidence [Internet]. PEN: Practice-based Evidence in Nutrition. 2019 [cited 2020 Dec 17]. Available from: https://www.pennutrition.com/KnowledgePathway.aspx?kpid=803&trid=8762&trcatid=42.

[3] Nguyen-Michel ST, Unger JB, Hamilton J, Spruijt-Metz D (2006). Associations between physical activity and perceived stress/hassles in college students. Stress Health.

[4] Stubbs B, Koyanagi A, Hallgren M, Firth J, Richards J, Schuch F (2017). Physical activity and anxiety: A perspective from the World Health Survey. J Affect Disord.

[5] Kalmpourtzidou A, Eilander A, Talsma EF (2020). Global Vegetable Intake and Supply Compared to Recommendations: A Systematic Review. Nutrients.

[6] Tugault-Lafleur CN, Black JL (2019). Differences in the Quantity and Types of Foods and Beverages Consumed by Canadians between 2004 and 2015. Nutrients.

[7] Noncommunicable diseases: Risk factors [Internet]. World Health Organization. 2022 [cited 2022 Apr 4]. Available from: https://www.who.int/data/gho/data/themes/topics/topic-details/GHO/ncd-risk-factors.

[8] World Health Organization. Global action plan on physical activity 2018–2030: more active people for a healthier world [Internet]. World Health Organization; 2018 [cited 2022 Apr 4]. 101 p. Available from: https://apps.who.int/iris/handle/10665/272722.

[9] Loyen A, Clarke-Cornwell AM, Anderssen SA, Hagströmer M, Sardinha LB, Sundquist K (2017). Sedentary Time and Physical Activity Surveillance Through Accelerometer Pooling in Four European Countries. Sports Med Auckl NZ.

[10] Guthold R, Stevens GA, Riley LM, Bull FC (2018). Worldwide trends in insufficient physical activity from 2001 to 2016: a pooled analysis of 358 population-based surveys with 1·9 million participants. Lancet Glob Health.

[11] Michie S, Marques MM, Norris E, Johnston M. Theories and Interventions in Health Behavior Change. In: T. A. Revenson, & R. A. R. Gurung (Eds.), Handbook of health psychology. Oxfordshire: Routledge; 2018. p. 69–88.

[12] Golden SD, Earp JAL (2012). Social ecological approaches to individuals and their contexts: twenty years of health education & behavior health promotion interventions. Health Educ Behav Off Publ Soc Public Health Educ.

[13] Foster RG (2020). Sleep, circadian rhythms and health. Interface Focus.

[14] Michie S, West R, Finnerty AN, Norris E, Wright AJ, Marques MM (2020). Representation of behaviour change interventions and their evaluation: Development of the Upper Level of the Behaviour Change Intervention Ontology. Wellcome Open Res.

[15] Cooper J, Murphy J, Woods C, Van Nassau F, McGrath A, Callaghan D (2021). Barriers and facilitators to implementing community-based physical activity interventions: a qualitative systematic review. Int J Behav Nutr Phys Act..

[16] Czajkowski SM, Hunter CM (2021). From ideas to interventions: A review and comparison of frameworks used in early phase behavioral translation research. Health Psychol.

[17] Gourlan M, Bernard P, Bortolon C, Romain Aj, Lareyre O, Carayol M (2016). Efficacy of theory-based interventions to promote physical activity. A meta-analysis of randomised controlled trials. Health Psychol Rev..

[18] Ashton LM, Sharkey T, Whatnall MC, Williams RL, Bezzina A, Aguiar EJ (2019). Effectiveness of Interventions and Behaviour Change Techniques for Improving Dietary Intake in Young Adults: A Systematic Review and Meta-Analysis of RCTs. Nutrients.

[19] Sremanakova J, Sowerbutts AM, Todd C, Cooke R, Burden S (2021). Systematic Review of Behaviour Change Theories Implementation in Dietary Interventions for People Who Have Survived Cancer. Nutrients.

[20] Hoffmann TC, Glasziou PP, Boutron I, Milne R, Perera R, Moher D (2014). Better reporting of interventions: template for intervention description and replication (TIDieR) checklist and guide. BMJ.

[21] Campbell KL, Van Patten CL, Neil SE, Kirkham AA, Gotay CC, Gelmon KA (2012). Feasibility of a Lifestyle Intervention on Body Weight and Serum Biomarkers in Breast Cancer Survivors with Overweight and Obesity. J Acad Nutr Diet.

[22] Park CL, Cho D, Salner AL, Dornelas E (2016). A Randomized Controlled Trial of Two Mail-based Lifestyle Interventions for Breast Cancer Survivors. Support Care Cancer Off J Multinatl Assoc Support Care Cancer.

[23] Reeves M, Winkler E, Mccarthy N, Lawler S, Terranova C, Hayes S (2017). The Living Well after Breast Cancer™ Pilot Trial: a weight loss intervention for women following treatment for breast cancer. Asia Pac J Clin Oncol.

[24] White JL, Ransdell LB (2003). Worksite intervention model for facilitating changes in physical activity, fitness, and psychological parameters. Percept Mot Skills.

[25] Liberati A, Altman DG, Tetzlaff J, Mulrow C, Gøtzsche PC, Ioannidis JPA (2009). The PRISMA statement for reporting systematic reviews and meta-analyses of studies that evaluate healthcare interventions: explanation and elaboration. BMJ.

[26] Salam RA, Das JK, Lassi ZS, Bhutta ZA (2016). Adolescent Health Interventions: Conclusions, Evidence Gaps, and Research Priorities. J Adolesc Health.

[27] Abbott P, Davison J, Moore L, Rubinstein R (2010). Barriers and enhancers to dietary behaviour change for Aboriginal people attending a diabetes cooking course. Health Promot J Aust Off J Aust Assoc Health Promot Prof.

[28] Astbury NM, Albury C, Nourse R, Jebb SA (2020). Participant experiences of a low-energy total diet replacement programme: A descriptive qualitative study. PLoS ONE.

[29] Burke LE, Swigart V, Warziski Turk M, Derro N, Ewing LJ (2009). Experiences of self-monitoring: successes and struggles during treatment for weight loss. Qual Health Res.

[30] Dasgupta K, Jarvandi S, De Civita M, Pillay S, Hajna S, Gougeon R (2014). Participants’ perceptions of a group based program incorporating hands-on meal preparation and pedometer-based self-monitoring in type 2 diabetes. PLoS ONE.

[31] Hammarström A, Wiklund AF, Lindahl B, Larsson C, Ahlgren C (2014). Experiences of barriers and facilitators to weight-loss in a diet intervention - a qualitative study of women in northern Sweden. BMC Womens Health.

[32] Maston G, Franklin J, Hocking S, Swinbourne J, Gibson A, Manson E (2021). Dietary adherence and program attrition during a severely energy-restricted diet among people with complex class III obesity: A qualitative exploration. PLoS ONE.

[33] Mendonça R de D, Guimarães LMF, Mingoti SA, Magalhães KA, Lopes ACS (2019). Barriers to and facilitators for adherence to nutritional intervention: Consumption of fruits and vegetables. Nutrition.

[34] Metzgar CJ, Preston AG, Miller DL, Nickols-Richardson SM (2015). Facilitators and barriers to weight loss and weight loss maintenance: a qualitative exploration. J Hum Nutr Diet Off J Br Diet Assoc.

[35] Rehackova L, Rodrigues AM, Thom G, Brosnahan N, Barnes AC, McCombie L (2022). Participant experiences in the Diabetes REmission Clinical Trial (DiRECT). Diabet Med J Br Diabet Assoc.

[36] Rodriguez R, Quintanilla C (2020). An exploratory study of factors motivating Mexican women to undertake and continue with nutritional programs. Health Mark Q.

[37] Camhi SM, Debordes-Jackson G, Andrews J, Wright J, Lindsay AC, Troped PJ (2021). Socioecological Factors Associated with an Urban Exercise Prescription Program for Under-Resourced Women: A Mixed Methods Community-Engaged Research Project. Int J Environ Res Public Health.

[38] Casey D, De Civita M, Dasgupta K (2010). Understanding physical activity facilitators and barriers during and following a supervised exercise programme in Type 2 diabetes: a qualitative study. Diabet Med J Br Diabet Assoc.

[39] Cooke AB, Pace R, Chan D, Rosenberg E, Dasgupta K, Daskalopoulou SS (2018). A qualitative evaluation of a physician-delivered pedometer-based step count prescription strategy with insight from participants and treating physicians. Diabetes Res Clin Pract.

[40] Gallegos-Carrillo K, Reyes-Morales H, Pelcastre-Villafuerte B, García-Peña C, Lobelo F, Salmeron J (2021). Understanding adherence of hypertensive patients in Mexico to an exercise-referral scheme for increasing physical activity. Health Promot Int.

[41] McCormack GR, McFadden K, McHugh TLF, Spence JC, Mummery K (2019). Barriers and facilitators impacting the experiences of adults participating in an internet-facilitated pedometer intervention. Psychol Sport Exerc.

[42] Eynon MJ, O’Donnell C, Williams L (2018). Gaining qualitative insight into the subjective experiences of adherers to an exercise referral scheme: A thematic analysis. J Health Psychol.

[43] Huberty JL, Ransdell LB, Sidman C, Flohr JA, Shultz B, Grosshans O (2008). Explaining long-term exercise adherence in women who complete a structured exercise program. Res Q Exerc Sport.

[44] Kinnafick FE, Thøgersen-Ntoumani C, Shepherd SO, Wilson OJ, Wagenmakers AJM, Shaw CS (2018). In It Together: A Qualitative Evaluation of Participant Experiences of a 10-Week, Group-Based, Workplace HIIT Program for Insufficiently Active Adults. J Sport Exerc Psychol.

[45] Korkiakangas EE, Alahuhta MA, Husman PM, Keinänen-Kiukaanniemi S, Taanila AM, Laitinen JH (2011). Motivators and barriers to exercise among adults with a high risk of type 2 diabetes–a qualitative study. Scand J Caring Sci.

[46] Lee H, Wilbur J, Chae D, Lee K, Lee M (2015). Barriers to performing stretching exercises among Korean-Chinese female migrant workers in Korea. Public Health Nurs Boston Mass.

[47] O’Dougherty M, Dallman A, Turcotte L, Patterson J, Napolitano MA, Schmitz KH (2008). Barriers and motivators for strength training among women of color and Caucasian women. Women Health.

[48] Tulloch H, Sweet SN, Fortier M, Capstick G, Kenny GP, Sigal RJ (2013). Exercise facilitators and barriers from adoption to maintenance in the diabetes aerobic and resistance exercise trial. Can J Diabetes.

[49] Vetrovsky T, Vetrovska K, Bunc V (2019). A qualitative exploration of the experiences of primary care patients engaged in email counseling meant to increase physical activity. Acta Gymnica.

[50] Viljoen JE, Christie CJA (2015). The change in motivating factors influencing commencement, adherence and retention to a supervised resistance training programme in previously sedentary post-menopausal women: a prospective cohort study. BMC Public Health.

[51] Brandt CJ, Clemensen J, Nielsen JB, Søndergaard J (2018). Drivers for successful long-term lifestyle change, the role of e-health: a qualitative interview study. BMJ Open.

[52] Chan RSM, Lok KYW, Sea MMM, Woo J (2009). Clients’ Experiences of a Community Based Lifestyle Modification Program: A Qualitative Study. Int J Environ Res Public Health.

[53] Hardcastle S, Hagger MS (2011). “You Can’t Do It on Your Own”: Experiences of a motivational interviewing intervention on physical activity and dietary behaviour. Psychol Sport Exerc.

[54] Harrison CR, Phimphasone-Brady P, DiOrio B, Raghuanath SG, Bright R, Ritchie ND (2020). Barriers and Facilitators of National Diabetes Prevention Program Engagement Among Women of Childbearing Age: A Qualitative Study. Diabetes Educ.

[55] Kleine HD, McCormack LA, Drooger A, Meendering JR (2019). Barriers to and Facilitators of Weight Management in Adults Using a Meal Replacement Program That Includes Health Coaching. J Prim Care Community Health.

[56] Leung AWY, Chan RSM, Sea MMM, Woo J. Psychological Factors of Long-Term Dietary and Physical Activity Adherence among Chinese Adults with Overweight and Obesity in a Community-Based Lifestyle Modification Program: A Mixed-Method Study. Nutrients [Internet]. 2020 May [cited 2022 Apr 4];12(5). Available from: http://www.ncbi.nlm.nih.gov/pmc/articles/PMC7284498/. 10.3390/nu12051379 PMC7284498 32408509

[57] Lieffers JRL, Quintanilha M, Trottier CF, Johnson ST, Mota JF, Prado CM (2021). Experiences with and Perception of a Web-Based Mindfulness, Nutrition, and Fitness Platform Reported by First-Year University Students: A Qualitative Study. J Acad Nutr Diet.

[58] Penn L, Dombrowski SU, Sniehotta FF, White M (2013). Participants’ perspectives on making and maintaining behavioural changes in a lifestyle intervention for type 2 diabetes prevention: a qualitative study using the theory domain framework. BMJ Open.

[59] Rise MB, Pellerud A, Rygg LØ, Steinsbekk A (2013). Making and maintaining lifestyle changes after participating in group based type 2 diabetes self-management educations: a qualitative study. PLoS ONE.

[60] Schmidt SK, Hemmestad L, MacDonald CS, Langberg H, Valentiner LS (2020). Motivation and Barriers to Maintaining Lifestyle Changes in Patients with Type 2 Diabetes after an Intensive Lifestyle Intervention (The U-TURN Trial): A Longitudinal Qualitative Study. Int J Environ Res Public Health.

[61] Wycherley TP, Mohr P, Noakes M, Clifton PM, Brinkworth GD (2012). Self-reported facilitators of, and impediments to maintenance of healthy lifestyle behaviours following a supervised research-based lifestyle intervention programme in patients with type 2 diabetes. Diabet Med J Br Diabet Assoc.

[62] Michie S, Richardson M, Johnston M, Abraham C, Francis J, Hardeman W (2013). The behavior change technique taxonomy (v1) of 93 hierarchically clustered techniques: building an international consensus for the reporting of behavior change interventions. Ann Behav Med Publ Soc Behav Med.

[63] Czajkowski SM, Powell LH, Adler N, Naar-King S, Reynolds KD, Hunter CM (2015). From Ideas to Efficacy: The ORBIT Model for Developing Behavioral Treatments for Chronic Diseases. Health Psychol Off J Div Health Psychol Am Psychol Assoc.

[64] CASP Qualitative Studies Checklist [Internet]. Critical Appraisal Skills Programme part of Oxford Centre for Triple Value Healthcare; 2019 [cited 2021 Jun 23]. Available from: https://casp-uk.b-cdn.net/wp-content/uploads/2018/03/CASP-Qualitative-Checklist-2018_fillable_form.pdf.

[65] Long HA, French DP, Brooks JM (2020). Optimising the value of the critical appraisal skills programme (CASP) tool for quality appraisal in qualitative evidence synthesis. Res Methods Med Health Sci.

[66] Teixeira PJ, Carraça EV, Marques MM, Rutter H, Oppert JM, De Bourdeaudhuij I (2015). Successful behavior change in obesity interventions in adults: a systematic review of self-regulation mediators. BMC Med.

[67] Locke EA, Latham GP (2002). Building a practically useful theory of goal setting and task motivation: A 35-year odyssey. Am Psychol.

[68] White JL, Ransdell LB, Vener J, Flohr JA (2005). Factors related to physical activity adherence in women: review and suggestions for future research. Women Health.

[69] Byrne M (2019). Increasing the impact of behavior change intervention research: Is there a role for stakeholder engagement?. Health Psychol Off J Div Health Psychol Am Psychol Assoc.

[70] World Cancer Research Fund & American Institute for Cancer Research. Diet, Nutrition, Physical Activity and Cancer: a Global Perspective. 3rd Ed. Geneva: World Health Organization; 2018. p. 1–116.

[71] DiMatteo MR (2004). Social support and patient adherence to medical treatment: a meta-analysis. Health Psychol Off J Div Health Psychol Am Psychol Assoc.

[72] Mayberry LS, Osborn CY (2012). Family support, medication adherence, and glycemic control among adults with type 2 diabetes. Diabetes Care.

[73] Miller TA, DiMatteo MR (2013). Importance of family/social support and impact on adherence to diabetic therapy. Diabetes Metab Syndr Obes Targets Ther.

[74] Quintiliani L, Poulsen S, Sorensen G (2010). Healthy Eating Strategies in the Workplace. Int J Workplace Health Manag.

[75] LWC Ng, Malhotra R, Lai D, Tai ES, Østbye T (2015). Perceived barriers to and ideas for weight control interventions in Malay homemakers: results from focus groups. Asia Pac J Public Health.

[76] Ryan RM, Deci EL (2000). Self-determination theory and the facilitation of intrinsic motivation, social development, and well-being. Am Psychol.

[77] Powell BJ, Beidas RS, Lewis CC, Aarons GA, McMillen JC, Proctor EK (2017). Methods to Improve the Selection and Tailoring of Implementation Strategies. J Behav Health Serv Res.

[78] Appel LJ, Champagne CM, Harsha DW, Cooper LS, Obarzanek E, Elmer PJ (2003). Effects of comprehensive lifestyle modification on blood pressure control: main results of the PREMIER clinical trial. J Am Med Assoc.

[79] Hannum SM, Carson LA, Evans EM, Petr EL, Wharton CM, Bui L (2006). Use of packaged entrees as part of a weight-loss diet in overweight men: an 8-week randomized clinical trial. Diabetes Obes Metab.

[80] Rock CL, Flatt SW, Sherwood NE, Karanja N, Pakiz B, Thomson CA (2010). Effect of a free prepared meal and incentivized weight loss program on weight loss and weight loss maintenance in obese and overweight women: a randomized controlled trial. JAMA.

[81] Gorin AA, Raynor HA, Niemeier HM, Wing RR (2007). Home grocery delivery improves the household food environments of behavioral weight loss participants: Results of an 8-week pilot study. Int J Behav Nutr Phys Act.

[82] Gorin AA, Raynor HA, Fava J, Maguire K, Robichaud E, Trautvetter J (2013). Randomized controlled trial of a comprehensive home environment-focused weight loss program for adults. Health Psychol Off J Div Health Psychol Am Psychol Assoc.

[83] Beckenstein H, Slim M, Kim H, Plourde H, Kilgour R, Cohen TR (2021). Acceptability of a structured diet and exercise weight loss intervention in breast cancer survivors living with an overweight condition or obesity: A qualitative analysis. Cancer Rep Hoboken NJ.

[84] Ahern DK (2007). Challenges and opportunities of eHealth research. Am J Prev Med.

[85] Zhao J, Freeman B, Li M (2016). Can Mobile Phone Apps Influence People’s Health Behavior Change? An Evidence Review. J Med Internet Res.

[86] WHO Global Observatory for eHealth, World Health Organization. MHealth: new horizons for health through mobile technologies. [Internet]. Geneva: World Health Organization; 2011 [cited 2021 Feb 25]. Available from: http://www.who.int/goe/publications/goe_mhealth_web.pdf

[87] Schoeppe S, Alley S, Van Lippevelde W, Bray NA, Williams SL, Duncan MJ (2016). Efficacy of interventions that use apps to improve diet, physical activity and sedentary behaviour: a systematic review. Int J Behav Nutr Phys Act.

[88] Baumann FT, Bieck O, Oberste M, Kuhn R, Schmitt J, Wentrock S (2017). Sustainable impact of an individualized exercise program on physical activity level and fatigue syndrome on breast cancer patients in two German rehabilitation centers. Support Care Cancer Off J Multinatl Assoc Support Care Cancer.

[89] Bock BC, Marcus BH, Pinto BM, Forsyth LH (2001). Maintenance of physical activity following an individualized motivationally tailored intervention. Ann Behav Med Publ Soc Behav Med.

[90] Campbell MK, DeVellis BM, Strecher VJ, Ammerman AS, DeVellis RF, Sandler RS (1994). Improving dietary behavior: the effectiveness of tailored messages in primary care settings. Am J Public Health.

[91] Ravasco P, Monteiro-Grillo I, Camilo M (2012). Individualized nutrition intervention is of major benefit to colorectal cancer patients: long-term follow-up of a randomized controlled trial of nutritional therapy. Am J Clin Nutr.

[92] Robb KA, Power E, Kralj-Hans I, Atkin WS, Wardle J (2010). The impact of individually-tailored lifestyle advice in the colorectal cancer screening context: a randomised pilot study in North-West London. Prev Med.

[93] Shilts MK, Horowitz M, Townsend MS (2004). Goal setting as a strategy for dietary and physical activity behavior change: a review of the literature. Am J Health Promot AJHP.

[94] Strecher VJ, Seijts GH, Kok GJ, Latham GP, Glasgow R, DeVellis B (1995). Goal setting as a strategy for health behavior change. Health Educ Q.

[95] Bodenheimer T, Handley MA (2009). Goal-setting for behavior change in primary care: an exploration and status report. Patient Educ Couns.

[96] Burke LE, Conroy MB, Sereika SM, Elci OU, Styn MA, Acharya SD (2011). The Effect of Electronic Self-Monitoring on Weight Loss and Dietary Intake: A Randomized Behavioral Weight Loss Trial. Obes Silver Spring Md.

[97] Carels RA, Darby LA, Rydin S, Douglass OM, Cacciapaglia HM, O’Brien WH (2005). The relationship between self-monitoring, outcome expectancies, difficulties with eating and exercise, and physical activity and weight loss treatment outcomes. Ann Behav Med.

[98] Turner-McGrievy GM, Beets MW, Moore JB, Kaczynski AT, Barr-Anderson DJ, Tate DF (2013). Comparison of traditional versus mobile app self-monitoring of physical activity and dietary intake among overweight adults participating in an mHealth weight loss program. J Am Med Inform Assoc JAMIA.

[99] Burnette JL, O’Boyle EH, VanEpps EM, Pollack JM, Finkel EJ (2013). Mind-sets matter: a meta-analytic review of implicit theories and self-regulation. Psychol Bull.

[100] O’Donnell S, Greene GW, Blissmer B (2014). The effect of goal setting on fruit and vegetable consumption and physical activity level in a Web-based intervention. J Nutr Educ Behav.

